# The risk of asthma in singletons conceived by ART: a retrospective cohort study

**DOI:** 10.1093/hropen/hoae041

**Published:** 2024-06-19

**Authors:** Shuangying Liu, Xiaoqian Zhou, Wei Wang, Min Zhang, Yu Sun, Xiaoling Hu, Jiali You, Xiaofei Huang, Yingzhi Yang, Guofang Feng, Lanfeng Xing, Long Bai, Minyue Tang, Yimin Zhu

**Affiliations:** Department of Reproductive Endocrinology, Women’s Hospital, School of Medicine, Zhejiang University, Hangzhou, China; Key Laboratory of Reproductive Genetics, Ministry of Education, Women’s Hospital, Zhejiang University School of Medicine, Hangzhou, China; Women’s Reproductive Health Laboratory of Zhejiang Province, Women’s Hospital, Zhejiang University School of Medicine, Hangzhou, China; Department of Reproductive Endocrinology, Women’s Hospital, School of Medicine, Zhejiang University, Hangzhou, China; Department of Reproductive Endocrinology, Women’s Hospital, School of Medicine, Zhejiang University, Hangzhou, China; Key Laboratory of Reproductive Genetics, Ministry of Education, Women’s Hospital, Zhejiang University School of Medicine, Hangzhou, China; Women’s Reproductive Health Laboratory of Zhejiang Province, Women’s Hospital, Zhejiang University School of Medicine, Hangzhou, China; Department of Reproductive Endocrinology, Women’s Hospital, School of Medicine, Zhejiang University, Hangzhou, China; Key Laboratory of Reproductive Genetics, Ministry of Education, Women’s Hospital, Zhejiang University School of Medicine, Hangzhou, China; Women’s Reproductive Health Laboratory of Zhejiang Province, Women’s Hospital, Zhejiang University School of Medicine, Hangzhou, China; Department of Reproductive Endocrinology, Women’s Hospital, School of Medicine, Zhejiang University, Hangzhou, China; Department of Reproductive Endocrinology, Women’s Hospital, School of Medicine, Zhejiang University, Hangzhou, China; Department of Reproductive Endocrinology, Women’s Hospital, School of Medicine, Zhejiang University, Hangzhou, China; Key Laboratory of Reproductive Genetics, Ministry of Education, Women’s Hospital, Zhejiang University School of Medicine, Hangzhou, China; Women’s Reproductive Health Laboratory of Zhejiang Province, Women’s Hospital, Zhejiang University School of Medicine, Hangzhou, China; Department of Reproductive Endocrinology, Women’s Hospital, School of Medicine, Zhejiang University, Hangzhou, China; Department of Reproductive Endocrinology, Women’s Hospital, School of Medicine, Zhejiang University, Hangzhou, China; Department of Reproductive Endocrinology, Women’s Hospital, School of Medicine, Zhejiang University, Hangzhou, China; Key Laboratory of Reproductive Genetics, Ministry of Education, Women’s Hospital, Zhejiang University School of Medicine, Hangzhou, China; Women’s Reproductive Health Laboratory of Zhejiang Province, Women’s Hospital, Zhejiang University School of Medicine, Hangzhou, China; Department of Reproductive Endocrinology, Women’s Hospital, School of Medicine, Zhejiang University, Hangzhou, China; Department of Reproductive Endocrinology, Women’s Hospital, School of Medicine, Zhejiang University, Hangzhou, China; Key Laboratory of Reproductive Genetics, Ministry of Education, Women’s Hospital, Zhejiang University School of Medicine, Hangzhou, China; Women’s Reproductive Health Laboratory of Zhejiang Province, Women’s Hospital, Zhejiang University School of Medicine, Hangzhou, China; Department of Reproductive Endocrinology, Women’s Hospital, School of Medicine, Zhejiang University, Hangzhou, China; Key Laboratory of Reproductive Genetics, Ministry of Education, Women’s Hospital, Zhejiang University School of Medicine, Hangzhou, China; Women’s Reproductive Health Laboratory of Zhejiang Province, Women’s Hospital, Zhejiang University School of Medicine, Hangzhou, China; Department of Reproductive Endocrinology, Women’s Hospital, School of Medicine, Zhejiang University, Hangzhou, China; Key Laboratory of Reproductive Genetics, Ministry of Education, Women’s Hospital, Zhejiang University School of Medicine, Hangzhou, China; Women’s Reproductive Health Laboratory of Zhejiang Province, Women’s Hospital, Zhejiang University School of Medicine, Hangzhou, China

**Keywords:** assisted reproduction, child follow-up, infertility, IVF/ICSI outcome, embryo transfer, ART safety

## Abstract

**STUDY QUESTION:**

Do singleton children conceived by ART have a higher asthma risk than naturally conceived (NC) singletons?

**SUMMARY ANSWER:**

The asthma risk was similar for ART-conceived singletons and NC singletons, and there were no clear differences between the various types of ART.

**WHAT IS KNOWN ALREADY:**

Whether ART increases asthma risk in offspring is questionable. The evidence is inconsistent and limited by ethnicity, geographic distribution, inadequate confounder adjustment, unsatisfactory control groups, and specific methods of ART. Furthermore, the mediating effects of obstetric and neonatal outcomes on the association between ART and asthma remain unclear.

**STUDY DESIGN, SIZE, DURATION:**

This observational, single-centre study was conducted at a reproductive centre of an affiliated university hospital between September 2009 and April 2023. A total of 3227 singletons aged 3–6 years conceived by IVF versus ICSI or fresh versus frozen embryo transfer were retrospectively enrolled, and a total of 1206 NC singletons of the same age were subsequently recruited.

**PARTICIPANTS/MATERIALS, SETTING, METHODS:**

Asthma was defined as a self-reported physician diagnosis or wheezing in the past 12 months. We performed multivariable logistic regression analyses to examine associations between asthma in offspring and ART use, adjusting for parental characteristics (age, education level, occupation type, BMI, asthma), smoking exposure, residence type, child sex, child age, and year of follow-up. Mediating effects were explored using longitudinal mediation structural equation modelling.

**MAIN RESULTS AND THE ROLE OF CHANCE:**

Asthma was reported for 51 (4.2%) of the 1206 NC singletons (median [interquartile range] age 5 [4–5] years; 48.1% females) and 169 (5.2%) of the 3227 ART-conceived singletons (5 [5–5] years; 47.6% females). We found that risks of childhood asthma in singletons conceived by ART were, overall, similar to those of NC singletons before (odds ratio [OR], 1.25 [95% CI, 0.92–1.74]; *P *=* *0.170) and after adjustment (adjusted OR [aOR], 0.66 [95% CI, 0.44–1.03]; *P *=* *0.126). The results were similar in multiple sensitivity analyses, and there were no clear differences in asthma risks according to the method of ART. Mediation analysis revealed a significant positive indirect effect of neonatal intensive care unit (NICU) admission (standard path coefficient, *b* = 0.025, *P *<* *0.05) and a negative indirect effect of breastfeeding (*b *= –0.012, *P *<* *0.05) on the association between ART and asthma in singleton offspring.

**LIMITATIONS, REASONS FOR CAUTION:**

This study is limited to singletons only and cannot be generalized. The study is also limited by its retrospective observational single-centre nature and sample size. Mediation analyses were exploratory. Therefore, the findings need to be interpreted with caution.

**WIDER IMPLICATIONS OF THE FINDINGS:**

These findings can help infertile couples undergoing ART be reassured about the risk of childhood asthma in singleton offspring. Breastfeeding is recommended as a potentially feasible intervention to reduce the asthma risks in ART-conceived children who are at increased potential risk of asthma, such as those with NICU admissions.

**STUDY FUNDING/COMPETING INTEREST(S):**

This work was supported by the Key Research and Development Program of Zhejiang Province (2021C03100), the National Key Research and Development Program of China (2021YFC2700603), and the Program for Key Subjects of Zhejiang Province in Medicine and Hygiene to Y. Z., the Zhejiang Province Natural Science Foundation (No. LQ22H040006) and the National Natural Science Foundation of China (No.82101759) to M.T., and the National Natural Science Foundation of China (No. 82201860) to J.Y. The authors declare no competing interests.

**TRIAL REGISTRATION NUMBER:**

ChiCTR2300069906.

WHAT DOES THIS MEAN FOR PATIENTS? Whether assisted reproductive technology (ART) increases the asthma risk in offspring has been questioned because of inconsistent evidence. This study was conducted in a group of Chinese women who had just one baby born (a singleton) when the offspring were 3–6 years of age. We found similar asthma risks for ART-conceived singletons and naturally conceived (NC) singletons, and there were no clear differences in asthma risk according to the method of ART used (IVF, ICSI, frozen or fresh embryo transfer). Patients undergoing ART should therefore be reassured about the risk of childhood asthma in offspring when applying elective single embryo transfer. Breastfeeding is recommended as a potentially feasible intervention to reduce the risk of asthma in ART-conceived children with pregnancy and neonatal complications.

## Introduction

Infertility is estimated to affect 186 million people worldwide (Inhorn and Patrizio, 2015) and 15.5% of couples in China (Zhou *et al.*, 2018). ART is an essential infertility treatment; however, concern arises that ART might pose risks to the long-term health of offspring, including asthma. The potential underlying mechanisms remain poorly understood but may involve specific ART procedures, epigenetic alterations (Novakovic *et al.*, 2019; Chen *et al.*, 2020; Schaub *et al.*, 2024), obstetric complications, and neonatal morbidity, which could act as mediators (Pandey *et al.*, 2012; Kallen *et al.*, 2013; Berntsen *et al.*, 2019), as well as intrinsic genetic factors in couples experiencing infertility (Carson *et al.*, 2013; Berntsen et al, 2019).

Asthma affects ∼645 million people globally (Song *et al.*, 2022). Asthma is a heterogeneous disease that tends to develop in early childhood (GBD Chronic Respiratory Disease Collaborators, 2020; Hammad and Lambrecht, 2021). However, whether ART increases asthma risk in offspring is questionable, because of the supporting (Kallen *et al.* 2005; Finnstrom *et al.*, 2011; Carson *et al.*, 2013; Kallen *et al.* 2013; Magnus *et al.*, 2019; Tsabouri *et al.*, 2021; Wijs *et al.*, 2021; Polinski *et al.*, 2022) and opposing (Halliday *et al.*, 2019; Kuiper *et al.*, 2019; Wijs *et al.*, 2022) findings. The evidence is limited by exclusive ethnicity and geographic distribution in developed countries, inadequate confounder adjustment, unsatisfactory control groups, and specific methods of ART. Furthermore, there might be concealed underlying mediating effects leading to an increased risk of asthma in ART-conceived children.

The aim of our study was to investigate whether there was an increased risk of childhood asthma after ART conception overall or after specific methods (IVF, ICSI, fresh embryo transfer [ET] and frozen embryo transfer [FET]). Further, we conducted a longitudinal mediation structural equation model (SEM) to explore the mediating effects of ART on childhood asthma.

## Materials and methods

### Setting and participants

**Figure 1.**
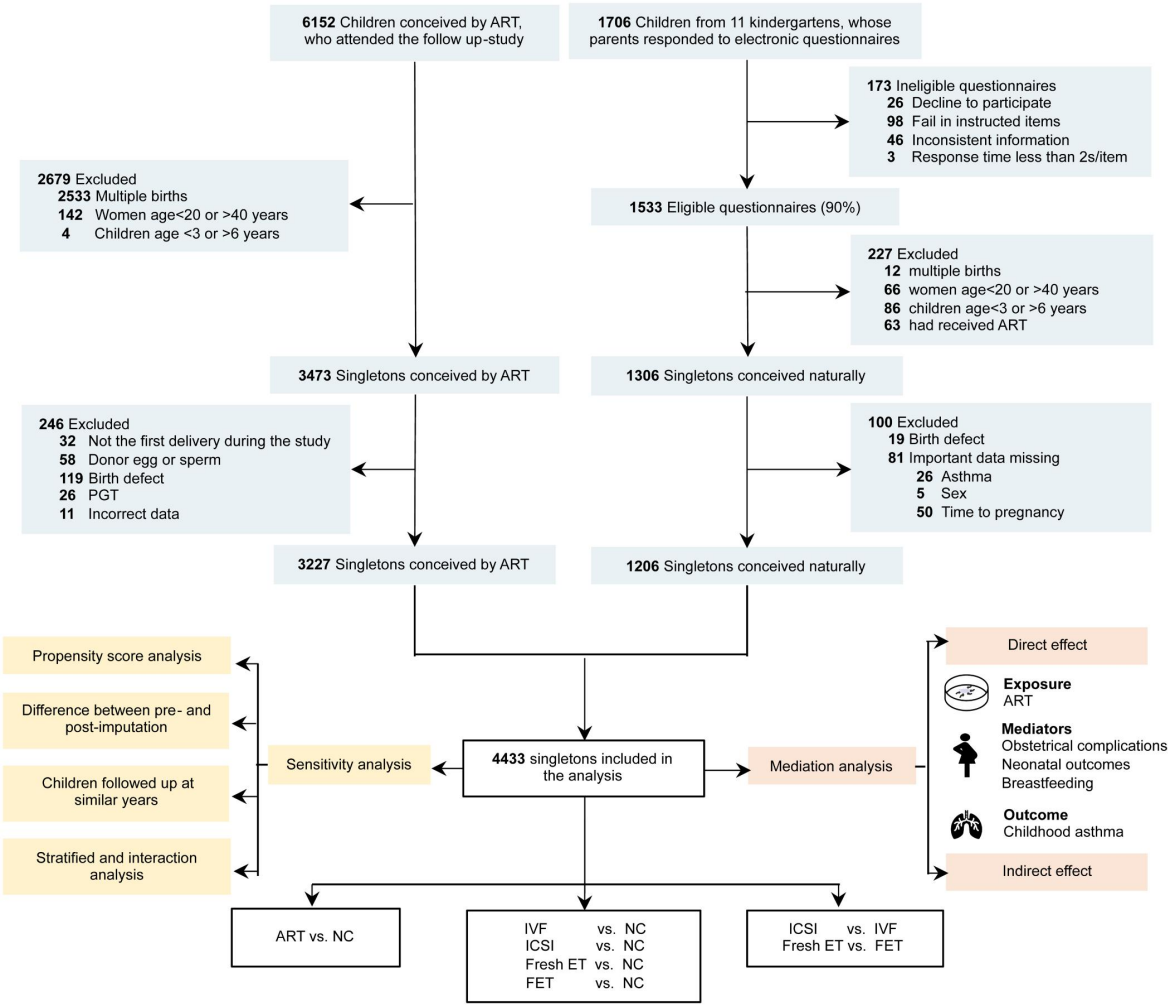
**Flowchart of the study of risk of asthma in singletons conceived by ART.** ET, embryo transfer; FET, frozen embryo transfer; NC, naturally conceived; PGT, preimplantation genetic testing.

We conducted this study at the Women’s Hospital, School of Medicine, Zhejiang University in Hangzhou, Zhejiang Province, China. A total of 6152 live-born children whose mothers initially underwent ART between December 2003 and February 2018 participated in the follow-up study at the ART Offspring Health Monitoring Center between September 2009 and April 2023 (Fig. 1). We restricted the study participants to singletons, given the low percentage of multiple pregnancies in natural pregnancies, which prevented us from obtaining an adequate control group. A total of 3227 singletons were included in this study after excluding multiples (n = 2533), mothers aged <20 or >40 years (n = 142), singletons aged <3 or >6 years (n = 4), not the first delivery of mothers who gave multiple births during the study period (n = 32), those conceived using donor eggs or sperm (n = 58), those with birth defects (n = 119), those for whom preimplantation genetic testing was performed (n = 26) and those with conflicting data (n = 11).

For naturally conceived (NC) children, 1706 children were recruited from 11 kindergartens whose parents responded to the questionnaires. After excluding children with ineligible questionnaires (n = 173), multiples (n = 12), maternal age <20 or >40 years (n = 66), singletons aged <3 or > 6 years (n = 86), those conceived by ART (n = 63), those with birth defects (n = 19), and those with missing important data (n = 81), a final sample of 1206 was obtained.

The study was performed with ethical approval granted by the Ethics Committee of the Women’s Hospital, School of Medicine, Zhejiang University (IRB-20220374-R) and registered with the Chinese Clinical Trial Registry (ChiCTR2300069906).

### Data sources

We retrieved the data of ART-conceived offspring from the clinical data warehouse, which includes patients’ demographic information, clinical diagnoses, medical histories, and ET cycle records. During the children’s visits, we conducted face-to-face questionnaire interviews to obtain sociodemographic information and information on pregnancy complications, neonatal characteristics, and child outcomes. The NC offspring data were collected using an online standardized questionnaire. Informed consent was obtained prior to participation.

### Study exposure and outcome

The exposure of interest was ART (IVF/ICSI/fresh ET and FET). Asthma was defined as a self-reported physician diagnosis or wheezing in the past 12 months according to the Global Burden of Disease (GBD Chronic Respiratory Disease Collaborators, 2020).

### Confounders and mediators

Potential confounders and mediators were selected based on previous publications and were incorporated into a directed acyclic graph (DAG, Supplementary Fig. S1). Minimal sufficient confounder sets to be adjusted were determined by DAGitty and included parental age (at delivery, years), parental education level (<college, ≥college), parental occupation type (categorized into three categories based on the European Socioeconomic Classification [E-SeC] and former study (Berger *et al.*, 2019; Zhang *et al.*, 2022): more advantaged [E-SeC classes 1–3], middle [E-SeC classes 4–6], and less advantaged [E-SeC classes 7–9]), parental BMI (calculated by dividing weight in kilograms by height in metres squared), parental asthma (self-report of a physician’s diagnosis), smoking exposure (self-reported smoking by the mother, father, or other main caregivers of the child), residence type (rural or urban living based on the participants’ registered home addresses and Chinese administrative divisions), and infertility for estimating the total effect of ART on asthma risk in offspring. Given that infertility was observed almost exclusively in couples undergoing ART, infertility characteristics (duration of attempt to conceive [years in continuous] and previous live birth) were not adjusted in the main analysis but were explored in sensitivity analyses. Additionally, child population characteristics (child sex, age at follow-up, and year at follow-up) were adjusted to ensure balance between the groups.

The potential mediators included obstetric complications (self-reported physician diagnoses of any of the complications during pregnancy, labour, and delivery, such as gestational diabetes mellitus, hypertensive disorders of pregnancy, liver disorders in pregnancy, premature rupture of the membranes, placenta previa, placental abruption, ovarian hyperstimulation syndrome, etc.), caesarean delivery, preterm birth (<37 weeks gestation), low birthweight (birthweight <2500 g), neonatal jaundice, respiratory distress syndrome, and feeding patterns (artificial feeding, mixed feeding, or breastfeeding in the first 6 months of life). Specifically, BMI for age (categorized into four groups according to growth standards for Chinese children under 7 years of age (China NHCotPsRo, 2022): underweight [<–2 SD], normal weight [–2 SD≤ · <+1 SD], overweight [+1 SD≤ · <+2SD], and obese [≥+2 SD]) was regarded as a mediator due to the early life growth lag in ART-conceived offspring (Hann *et al.*, 2018; Elhakeem *et al.*, 2022). Mediators were not adjusted for to estimate the total effect of ART and were subsequently investigated in the mediation analysis.

### Statistical analysis

All analyses in this study were carried out using R software (version 4.2.3, R Foundation for Statistical Computing, Vienna, Austria). The χ^2^ test or Fisher’s exact test was used to determine differences between groups for categorical variables, and the Welch two-sample *t*-test was utilized for continuous variables. The data are presented as frequencies (percentages) for categorical variables and medians (interquartile ranges [IQRs] for non-normally distributed and standard deviations for continuous variables. A two-tailed *P* value of <0.05 was considered to indicate statistical significance.

Before analysis, baseline characteristics were examined for missing data, and the range of missing data proportions ranged from 0% to 8.36%. Missing data were then imputed using multiple imputations by chained equations with the default cases (*k*) = 10. The study size was determined by the available data.

Logistic regression models were utilized to estimate odds ratios (ORs) and 95% CIs for asthma in singleton children associated with ART. The initial multivariable logistic regression model was adjusted for parental age, parental education level, parental occupation type, parental BMI, parental asthma, smoking exposure, residence type, and child sex, age at follow-up, and year at follow-up. When comparing ICSI with IVF and comparing FET with fresh ET, the multivariable logistic regression models were additionally adjusted for parental infertility characteristics, including duration of attempt to conceive and previous live birth.

To address the potential biases that may arise from the nonrandomized treatment administration of ART, we further employed propensity score (PS) analysis in order to reduce the effects of confounding. PSs for ART treatment were estimated with the same confounders except for the year of follow-up. The first PS strategy used was inverse probability of treatment weighting (IPTW), in which children were weighted by the inverse of the probability of conception by ART. The second strategy applied was propensity-score matching (PSM) at a 1:1 ratio, employing the nearest neighbour strategy with a calliper width of 0.2 and no replacement. The third strategy involved adjusting the PS as an additional confounder.

Moreover, to evaluate the robustness of the results, we performed several sensitivity analyses. First, we compared the differences in missing variables before and after imputation to examine how imputation might have affected our results. Second, we restricted the study population to singletons followed up during 2020–2023 to minimize possible bias introduced by difference in follow-up. Logistic regression models were applied to the restricted population with or without adjustment for the same confounders, as described in the main analysis. Third, stratified and interaction analysis were conducted according to child sex, duration of attempt to conceive, and other parental characteristics (age, occupation type, education level, BMI, and asthma).

In the mediation analysis, the R package mediation was performed for each potential mediator between ART and asthma in offspring with adjustment for the same confounders as the initial multivariable logistic regression model. Indirect and direct effects with 95% CIs were calculated. The indirect effects reflect the pathway from ART to asthma in offspring through mediators, while the direct effects represent the pathway that is independent of the mediators. The potential mediators found to have significant indirect effects on associations of interest were fit in a SEM for further exploration.

## Results

### Characteristics of the study population

A total of 1206 NC singletons and 3227 ART-conceived singletons were included in the study. The median age (IQR) at follow-up was 5 (4–5) years for NC singletons and 5 (5–5) years for ART-conceived singletons, and the proportions of females were 48.1% and 47.6%, respectively. ART-conceived children differed from NC children regarding their parental baseline characteristics and had more obstetric complications, compromised perinatal outcomes, and a higher proportion of exclusive breastfeeding in the first 6 months (Table 1). After IPTW (Supplementary Table S1), the cohort included 4619.6 NC offspring and 4444.9 ART-conceived offspring, and the matched cohort included 981 offspring in each group. Groups were balanced regarding baseline confounders except for infertility characteristics after weighting/matching. The PSs before and after weighting/matching with standard mean differences across covariates are shown in Supplementary Fig. S2.

**Table 1:** Baseline characteristics of the ART-conceived singletons and singletons born following natural conception.

Characteristics	NC (n = 1206)	ART (n = 3227)	*P* Value^a^
Child sex, No. (%)			
Female	580 (48.1)	1535 (47.6)	0.781
Male	626 (51.9)	1692 (52.4)	
Age at follow-up (years), median (IQR)	5 (4- 5)	5 (5-5)	<0.001
Maternal age (years), median (IQR)	27 (24-31)	30 (28-33)	<0.001
Paternal age (years), median (IQR)	29 (26-33)	32 (30-35)	<0.001
Maternal education level, No. (%)			
<College	890 (73.8)	2439 (75.6)	0.237
≥College	316 (26.2)	788 (24.4)	
Paternal education level, No. (%)			
<College	898 (74.5)	2318 (71.8)	0.088
≥College	308 (25.5)	909 (28.2)	
Maternal occupation type, No. (%)			
Less advantage	477 (39.6)	1087 (33.7)	<0.001
Middle	520 (43.1)	1751 (54.3)	
Most advantaged	209 (17.3)	389 (12.1)	
Paternal occupation type, No. (%)			
Less advantage	253 (21.0)	422 (13.1)	<0.001
Middle	646 (53.6)	2449 (75.9)	
Most advantaged	307 (25.5)	356 (11.0)	
Maternal BMI, No. (%)			
Normal	792 (65.7)	2267 (70.3)	<0.001
Underweight	167 (13.8)	269 (8.3)	
Overweight	216 (17.9)	578 (17.9)	
Obesity	31 (2.6)	113 (3.5)	
Paternal BMI, No. (%)			
Normal	608 (50.4)	1728 (53.5)	0.200
Underweight	45 (3.7)	97 (3.0)	
Overweight	443 (36.7)	1105 (34.2)	
Obese	110 (9.1)	297 (9.2)	
Parental asthma, No. (%)	41 (3.4)	148 (4.6)	0.098
Smoking exposure, No. (%)	577 (47.8)	1000 (31.0)	<0.001
Residence, No. (%)			
Rural	901 (74.7)	1601 (49.6)	<0.001
Urban	305 (25.3)	1626 (50.4)	
Duration of attempt to conceive, median (IQR), years	0 (0-0)	3(2-5)	<0.001
Previous live birth, No. (%)	533 (44.2)	290 (9.0)	<0.001
Fertilization methods, No. (%)			
IVF	NA	2417 (74.9)	NA
ICSI	NA	810 (25.1)	
Embryo transfer methods, No. (%)			
Fresh	NA	1928 (59.7)	NA
Frozen	NA	1299 (40.3)	
Potential mediators			
Obstetric complications, No. (%)	474 (39.3)	1741 (54.0)	<0.001
Gestational diabetes mellitus, No. (%)	95 (7.9)	342 (10.6)	0.008
Hypertensive disorder of pregnancy, No. (%)	47 (3.9)	181 (5.6)	0.026
Liver disorders in pregnancy, No. (%)	22 (1.8)	46 (1.4)	0.410
Premature rupture of the membranes, No. (%)	14 (1.2)	377 (11.7)	<0.001
Respiratory distress syndrome, No. (%)	20 (1.7)	201 (6.2)	<0.001
Cesarean section, No. (%)	463 (38.4)	2564 (79.5)	<0.001
Preterm birth, No. (%)	80 (6.6)	275 (8.5)	0.046
Macrosomia, No. (%)	16 (1.3)	194 (6.0)	<0.001
Low birth weight, No. (%)	77 (6.4)	162 (5.0)	0.086
Neonatal intensive care unit admission, No. (%)	149 (12.4)	548 (17.0)	<0.001
Jaundice, No. (%)	92 (7.6)	528 (16.4)	<0.001
Feeding patterns,^b^ No. (%)			
Artificial only	201 (16.7)	590 (18.3)	<0.001
Mixed	463 (38.4)	845 (26.2)	
Breast only	542 (44.9)	1792 (55.5)	
BMI-for-age, No. (%)			
Normal	710 (58.9)	2598 (80.5)	<0.001
Underweight	114 (9.5)	144 (4.5)	
Overweight	232 (19.2)	359 (11.1)	
Obese	150 (12.4)	126 (3.9)	

IQR, interquartile range; NA, not available; NC, naturally conceived.

a *P* values were calculated using the χ^2^ test or Fisher exact test for categorical variables and the Mann–Whitney *U* tests for continuous variables.

b Feeding patterns in the first 6 months of life.

### ART versus NC and its sensitive analysis

As shown in Table 2, asthma was reported in 4.2% and 5.2% of the NC singletons and ART-conceived singletons, respectively. No significant differences were observed in asthma risk between ART-conceived and NC singletons in the crude (OR, 1.25; 95% CI, 0.92–1.74), multivariable (adjusted OR [aOR], 0.66; 95% CI, 0.44–1.03), IPTW (OR, 0.77; 95% CI, 0.41–1.42), and PSM analyses (OR, 0.84; 95% CI, 0.36–1.82), or after adjusting for PSs and year of follow-up (aOR, 0.64; 95% CI, 0.37–1.06).

**Table 2:** Association between ART and asthma in offspring.

Analysis	NC Asthma/total (%)	ART Asthma/total (%)	OR (95% CI)	*P* value^f^
Crude analysis	51/1206 (4.2)	169/3227 (5.2)	1.25 (0.92–1.74)	0.170
Multivariable analysis^a^	51/1206 (4.2)	169/3227 (5.2)	0.66 (0.44–1.03)	0.126
Propensity-score analysis^b^				
IPTW^c^	229.9/4619.6 (5.0)	229.0/4444.9 (5.2)	0.77 (0.41–1.42)	0.396
PSM^d^	42/981(4.3)	49/981 (5.0)	0.84 (0.36–1.82)	0.437
Adjusted for propensity score^e^	51/1206 (4.2)	169/3227 (5.2)	0.64 (0.37–1.06)	0.090

IPTW, inverse probability of treatment weighting; NC, naturally conceived; OR, odds ratio; PSM, propensity score matching.

a Multivariable analysis using logistics regression model adjusted for parental age (continuous), parental education level (categorical), parental BMI (categorical), parental occupation type (categorical), parental asthma (binary), smoking exposure (binary), residence type (binary), child sex (binary), age at follow-up (continuous), and year at follow-up (categorical).

b Confounders used in calculating propensity score were as same as the multivariable analysis except not including year at follow-up.

c Children with inverse probability of treatment weighting according to propensity score. Year at follow-up was additionally adjusted when estimating OR and 95% CI with logistics regression model.

d Children with matching according to the propensity score. Year at follow-up (categorical) was additionally adjusted when estimating OR and 95% CI with logistics regression model.

e OR and 95% CI were estimated with logistics regression model adjusted for propensity score and year at follow-up.

f Statistical significance was set at *P *<* *0.05.

To evaluate the robustness of the total effect of ART on childhood asthma, we performed several sensitivity analyses. First, no significant difference was found in missing variables before and after multiple imputations (Supplementary Table S2). Second, we restricted the study population to children who were followed up between 2020 and 2023. The baseline characteristics of the restricted population were quite similar to that of the primary population between ART-conceived and NC children, as shown in Supplementary Table S3. Similarly, no significant difference in childhood asthma was found between ART-conceived and NC children followed up during 2020–2023, before and after adjusting for parental age, parental education level, parental BMI, parental occupation type, parental asthma, smoking exposure, residence type, child age at follow-up, child sex and year of follow-up (Supplementary Table S4). Next, we performed interaction and stratified analyses (Supplementary Table S5). The results in subgroups were not significant, as in the main analysis regarding asthma in ART-conceived and NC offspring: between females and males, between parents with duration of attempt to conceive <1 year and >1 year, between parents aged <35 and ≥35 years, between parental education < college and ≥ college, between groups with or without parental asthma, and among groups with different parental occupations and BMIs.

### Specific ART (IVF/ICSI/fresh ET/FET) versus NC

We then investigated childhood asthma between NC singletons and singletons conceived by different methods of ART (Table 3). Asthma risk was found to be significantly higher in ICSI singletons than in NC singletons according to the crude analysis (OR, 1.58; 95% CI, 1.07–2.34), but could be adjusted for parental age, parental education, parental BMI, parental occupation type, parental asthma, smoking exposure, residence type, child sex, child age, and year of follow-up (aOR, 0.72; 95% CI, 0.40–1.26). The results indicated that the ORs of asthma were similar between IVF-conceived singletons and NC singletons. Furthermore, the ORs of asthma in the fresh ET-conceived singletons versus NC singletons were 1.40 (95% CI, 1.00-1.98) before and 0.66 (95% CI, 0.39-1.13) after adjustment. The ORs of asthma in the FET-conceived singletons versus NC singletons were 1.04 (95% CI, 0.71–1.53) before and 0.61 (95% CI, 0.39–1.04) after adjustment.

**Table 3:** Associations between ART methods and childhood asthma in singletons.

	IVF (n = 2417)	ICSI (n = 810)	Fresh ET (n = 1928)	FET (n = 1299)
Asthma, No (%)	116 (4.8)	53 (6.5)	112 (5.8)	57 (4.4)
Group versus NC				
OR (95% CI)^a^	1.14 (0.82–1.61)	**1.58 (1.07–2.34)**	1.40 (1.00–1.98)	1.04 (0.71–1.53)
aOR (95% CI)^b^	0.66 (0.44–1.02)	0.72 (0.40–1.26)	0.66 (0.39–1.13)	0.61 (0.39–1.04)
Group versus reference	ICSI versus IVF		FET versus fresh ET	
OR (95% CI)^a^	1.39 (0.99–1.93)		0.74 (0.53–1.02)	
aOR (95% CI)^b^	1.37 (0.97–1.92)		0.72 (0.51–1.00)	
aOR (95% CI)^c^	1.37 (0.97–1.93)		0.77 (0.55–1.08)	

aOR, adjusted odds ratio; ET, embryo transfer; FET, frozen embryo transfer; NC, naturally conceived; OR, odds ratio; Numbers in bold denote statistically significant results.

a Logistic regression model without adjusting.

b Multivariable logistic regression model included parental age (continuous), parental education level (categorical), parental BMI (categorical), parental occupation type (categorical), parental asthma (binary), smoking exposure (binary), residence type (binary), child sex (binary), child age (continuous), and year of follow-up (categorical).

c Additionally adjusting for duration of attempt to conceive (continuous) and previous live birth (binary).

### Internal variance of ART (ICSI versus IVF or FET versus fresh ET)

We further investigated asthma in ART-conceived singletons by different fertilization or ET methods (Table 3). There was a small increase in risk of asthma in ICSI-conceived singletons compared to IVF-conceived singletons (OR, 1.39; 95% CI, 0.99–1.93) and decrease in risk of asthma in FET-conceived singletons compared with singletons conceived by fresh ET (OR, 0.74; 95% CI, 0.53–1.02). The aORs showed similar trends but did not reach significance (ICSI versus IVF: aOR, 1.37 [95% CI, 0.97–1.93]; FET versus fresh ET: aOR, 0.77 [95% CI, 0.55–1.08]).

### Mediation analysis

Mediation analyses were performed to evaluate the potential mediating effects of obstetric and neonatal outcomes on the associations between ART and childhood asthma risk in singleton offspring (Supplementary Table S6). Among the 14 potential mediators, obstetric complications, neonatal intensive care unit (NICU) admission, and feeding patterns had significant indirect effects on the associations of interest, after adjusting for parental age, parental education, parental BMI, parental occupation type, parental asthma, smoking exposure, residence type, child age at follow-up, child sex, and year of follow-up. The direct effects of ART on childhood asthma were not significant in any mediation analyses. The indirect effect estimates of obstetric complications, NICU admission, and feeding patterns were 0.29% (95% CI, 0.04–0.48%), 0.08 (95% CI, 0.01–0.26%), and –0.19 (95% CI, –0.30 to –0.03%), respectively.

**Figure 2.**
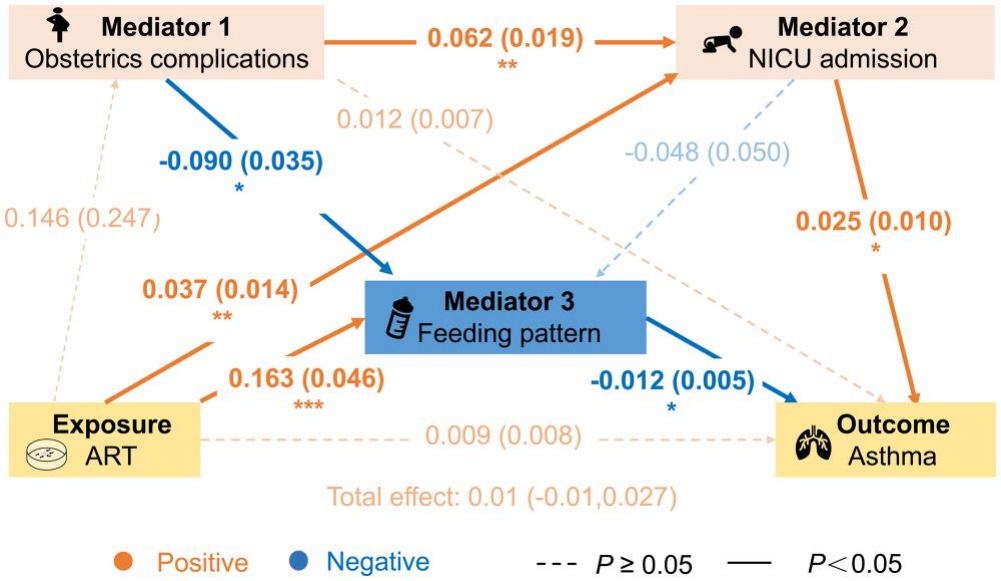
**Longitudinal mediation structural equation modelling of association between ART and childhood asthma**. Structural equation modelling analysis was performed with R software using the lavaan package (version 0.6-17; Rosseel, 2012). Values represent standard path coefficients with standard errors. Confounders are not included for simplicity. Orange and blue arrows indicate positive and negative relationships, respectively. Solid lines indicate statistically significant paths, while dashed lines indicate nonsignificant paths. NICU, neonatal intensive care unit. *P < 0.05, **P < 0.01, ***P < 0.001.

Furthermore, we conducted structural equation modelling to assess the concurrent effect of obstetric complications, NICU admission, and feeding patterns on the association between ART and subsequent childhood asthma, as displayed in Fig. 2. The resulting direct and total effects of ART on childhood asthma were not significant after accounting for all the paths. ART was associated with increased NICU admission risk (standard path coefficient, *b* = 0.037, *P *<* *0.01), and NCIU admission was associated with increased asthma risk in offspring (*b* = 0.025, *P *<* *0.05). However, ART was associated with higher breastfeeding proportions (*b* = 0.163, *P *<* *0.001), which, in turn, was associated with lower asthma risk in offspring (*b* = –0.012, *P *<* *0.05). Specifically, obstetric complications could increase asthma risk in offspring by positively affecting NICU admission (*b* = 0.062, *P *=* *0.001) and negatively affecting breastfeeding paths (*b* = –0.048). Therefore, the nonsignificant total effect of ART on childhood asthma may be a result of the opposing effects of the mediators.

## Discussion

### Principal findings

We found that risks of childhood asthma in Chinese singletons conceived by ART were overall similar to those of NC singletons. Regarding the fertilization methods, IVF-conceived singletons exhibited comparable asthma risks to NC singletons, while asthma risks were slightly elevated in ICSI-conceived singletons, but the results were not significant after adjustment for demographic characteristics. Similar asthma risks were observed between NC singletons and singletons conceived by ET or FET. The mediation analysis, further reinforcing the reassuring finding, indicated a nonsignificant total effect and direct effect of overall ART on asthma in singleton offspring. NICU admission and breastfeeding might play mediating roles in associations between ART and childhood asthma in singletons, with the opposite trend.

### Comparison with previous studies

Studies to date have been largely conducted in Europe (Ericson *et al.*, 2002; Bonduelle *et al.*, 2005; Kallen *et al.*, 2005; Finnstrom *et al.*, 2011; Carson *et al.*, 2013; Guibas *et al.*, 2013; Harju *et al.*, 2013; Kallen *et al.*, 2013; Kuiper *et al.*, 2015, 2019; Magnus *et al.*, 2019), Australia (Halliday *et al.*, 2019), and the USA (Sicignano *et al.*, 2010; Polinski *et al.*, 2022), with Caucasian individuals as the study group. Our study focused on Asian individuals, with notable disparities in race, ethnicities, geographic locations, and infertility characteristics of the study populations. In China, tubal infertility accounts for more than half of all infertility cases (Tzeng *et al.*, 2023), compared to around 13.9% among White women in the USA (Anyalechi *et al.*, 2021). Additionally, ICSI, originally employed to address severe male infertility, has supplanted IVF as the most prevalent fertilization method in many countries (Chambers *et al.*, 2021). Nevertheless, only 25% of ART-conceived singletons were fertilized by ICSI in our centre. Previous studies also revealed similarities (e.g*. IL1RL1*, *TSLP*, *GATA3)* and discrepancies (e.g*. PTGDR*, *CRHR2*, *HLA*) in the genetic variants between Asians and Caucasians (Leung *et al.*, 2014; An *et al.*, 2021). These clinical differences may result in discrepancies in the total impact of ART on offspring.

Two recent systematic reviews and meta-analyses (Tsabouri *et al.*, 2021; Wijs *et al.*, 2021) reported a significantly, yet modestly, elevated risk of asthma in ART-conceived children. However, former studies exhibited high heterogeneity owing to study design, confounder adjustment, and selection bias. As noted in the latest meta-analysis (Wijs *et al.*, 2021), the increased risk was no longer significant in the analysis when the sample included only singleton children, of the appropriate age (≥3 years), confirmed ART use, and an objective asthma diagnosis. This suggests that the evaluated risks of asthma in ART-conceived offspring compared to NC offspring may be attributed to the mediating effect of multiple pregnancies. The selection of singletons as the study population in our study allows us to elucidate the impact of ART on childhood asthma more purely, through ART itself rather than through multiple ET. Our findings that the asthma risks were similar between ART-conceived singletons and NC singletons were consistent with the latest meta-analysis (Wijs *et al.*, 2021) and other cohort studies (Halliday *et al.*, 2019; Kuiper *et al.*, 2019; Wijs *et al.*, 2022).

In addition, differences in changes of feeding patterns after ART could result in variations in asthma outcomes. In our results, there was a greater proportion of ART-conceived children who were breastfeeding, and there was no difference in subsequent asthma during preschool age. This may be attributed to the protective mediation effect of breastfeeding on the associations between ART and asthma, which acts in an opposite direction to obstetric complications and NICU admission. A multi-centre study in the USA, including New York state, reported a significant decline in breastfeeding at 8 weeks among women receiving ART, after adjusting for basic demographic covariates and maternal health conditions (Barrera *et al.*, 2019). Correspondingly, a prospective cohort study conducted in New York State revealed that ART-conceived children were at increased risk of childhood asthma and atopy (Polinski *et al.*, 2022). Although these two studies differ in terms of duration, the combined results indicate that the increase in childhood asthma risk is probably associated with the decrease in breastfeeding among women receiving ART. Nevertheless, few studies on asthma related to ART have analysed feeding patterns or mediation mechanisms (Ericson *et al.*, 2002; Bonduelle *et al.*, 2005; Sicignano *et al.*, 2010; Finnstrom *et al.*, 2011; Guibas *et al.*, 2013; Harju *et al.*, 2013; Kallen *et al.*, 2013; Kuiper *et al.*, 2015; Fruchter *et al.*, 2017; Magnus *et al.*, 2019; Polinski *et al.*, 2022; Wijs *et al.*, 2022).

Furthermore, our research extends prior studies in the exploration of associations between asthma and specific methods of ART. Most studies investigating the association between ART and asthma have focused solely on IVF (Ericson *et al.*, 2002; Kallen *et al.*, 2005; Sicignano *et al.*, 2010; Finnstrom *et al.*, 2011; Guibas *et al.*, 2013; Kallen *et al.*, 2013; Halliday *et al.*, 2019; Kuiper *et al.*, 2019) or combined IVF and ICSI (Carson *et al.*, 2013; Harju *et al.*, 2013; Kuiper *et al.*, 2015, 2019; Polinski *et al.*, 2022; Wijs *et al.*, 2022), with only a few studies comparing ICSI and IVF. An early multi-centre study found that rates of use of asthma medication were similar in NC singletons, IVF-conceived singletons, and ICSI-conceived singletons at the age of 5 years (Bonduelle *et al.*, 2005). A national study in Norway showed that the asthma risk in ICSI-conceived children (5.2%) was comparable to that in IVF-conceived children (5.1%) (Magnus *et al.*, 2019). These two studies were consistent with our findings in which we found no significant difference in asthma risk among NC singletons, IVF-conceived singletons, and ICSI-conceived singletons. We also noticed a trend towards an increased risk of asthma in ICSI-conceived singletons compared to IVF-conceived singletons, although this was not statistically significant. The cause of the observed trend remains unclear. The invasive fertilization procedure of ICSI may disrupt the ultrastructure of the oocyte, imposing changes in epigenetic modifications and a threat to offspring safety (Qiao *et al.*, 2010; Yang *et al.*, 2021). Other potential contributing factors may include unmeasured confounders and male factor infertility. Further investigation is required to ascertain the potential impact of ICSI on childhood asthma. Moreover, the risks of asthma in singletons conceived by fresh ET and FET have not been reported before. Our findings indicate a slightly lower risk of asthma in FET-conceived singletons compared to singletons conceived by fresh ET, which may be mediated by a decreased incidence of obstetric complications in mothers undergoing FET cycles. However, we cannot draw full conclusions as the results did not reach statistical significance.

### Implications

Asthma is one of the most prevalent diseases worldwide and currently lacks a cure, thus causing a serious burden on both families and society. In this study, we found an overall similar risk of childhood asthma in ART-conceived singletons and NC singletons, which should reassure couples undergoing ART. It was recommended that elective single ET be employed, as this not only reduces the risks of obstetric complications and NICU admissions, but also helps the mother to maintain adequate breastfeeding for the offspring. Breastfeeding may serve as a bio-behavioural mechanism that prevents ART-conceived children from having asthma. Doctors at reproductive centres should educate couples undergoing ART on the importance of breastfeeding, and promote a prolonged duration of exclusive breastfeeding to improve the health of the offspring. Apart from breastfeeding, research could be conducted on the impact of other feasible interventions for preventing asthma in ART-conceived children, such as reducing air pollution, encouraging a balanced diet and improving feto-maternal health (Beasley *et al.*, 2015). Moreover, given the potential trend towards an increased risk of asthma in ICSI-conceived singletons, routine use of ICSI is not recommended in infertile couples with a non-severe male factor.

### Strengths and limitations

The strengths of the study cohort include the recruitment and long-term follow-up of ART-conceived children whose parents had detailed medical records, the inclusion of an Asian population, and a relatively large number of ART-conceived children. Regarding statistical analysis, strengths include the use of DAG to determine confounders, the application of IPTW and PSM to minimize confounders, and the use of mediation analysis.

A major limitation of this study was that we only included singleton children, and there was a possibility that the impact of ART on childhood asthma may differ significantly between ART-conceived singletons and multiples. Besides, the ART-conceived and NC singletons were recruited from incompletely overlapping time periods. Therefore, we adjusted for the year of follow-up, and we conducted an additional sensitivity analysis of children who were followed up between 2020 and 2023, and any differences in the results were not significant. Second, the study size was determined by the available data, and some outcomes had low precision/power. Moreover, childhood asthma and several other variables were determined using self-reports, which may introduce misclassification. Furthermore, the results of the subgroup and sensitivity analyses should be considered as exploratory because of the potential for type I error. In addition, the results of mediation analysis should be interpreted with caution because of the weak fit indices in some models and the type I error.

## Conclusion

In this retrospective cohort study, we found that risk of childhood asthma in Chinese singletons conceived by ART was comparable to that of NC singletons. There were no clear differences in asthma risk according to specific fertilization or ET method of ART. Furthermore, mediation analysis revealed a significant positive indirect effect of NICU admission and a negative indirect effect of breastfeeding on the association between ART and asthma in singleton offspring. Overall, the results regarding childhood asthma after ART were reassuring, and we propose that breastfeeding is recommended for ART-conceived children who are at increased potential risk of asthma, such as those with NICU admissions.

## Supplementary Material

hoae041_Supplementary_Data

## Data Availability

The data underlying this article will be shared on reasonable request to the corresponding author.

## References

[hoae041-B1] An J , Do AR, Kang HY, Kim WJ, Lee S, Lee JH, Song WJ, Kwon HS, Cho YS, Moon HB et al Genome-wide association study of Korean asthmatics: a comparison with UK asthmatics. Allergy Asthma Immunol Res 2021;13:609–622. 34212547 10.4168/aair.2021.13.4.609 PMC8255356

[hoae041-B2] Anyalechi GE , Wiesenfeld HC, Kirkcaldy RD, Kissin DM, Haggerty CL, Hammond KR, Hook EW 3rd, Bernstein KT, Steinkampf MP, Geisler WM. Tubal factor infertility, in vitro fertilization, and racial disparities: a retrospective cohort in two US clinics. Sex Transm Dis 2021;48:748–753. 33833148 10.1097/OLQ.0000000000001435 PMC9012243

[hoae041-B3] Barrera CM , Kawwass JF, Boulet SL, Nelson JM, Perrine CG. Fertility treatment use and breastfeeding outcomes. Am J Obstet Gynecol 2019;220:261.e1–261.e7. 10.1016/j.ajog.2018.11.1100 PMC10983013 30513338

[hoae041-B4] Beasley R , Semprini A, Mitchell EA. Risk factors for asthma: is prevention possible? Lancet 2015;386:1075–1085. 26382999 10.1016/S0140-6736(15)00156-7

[hoae041-B5] Berger E , Castagne R, Chadeau-Hyam M, Bochud M, d’Errico A, Gandini M, Karimi M, Kivimaki M, Krogh V, Marmot M et al Multi-cohort study identifies social determinants of systemic inflammation over the life course. Nat Commun 2019;10:773. 30770820 10.1038/s41467-019-08732-x PMC6377676

[hoae041-B6] Berntsen S , Soderstrom-Anttila V, Wennerholm UB, Laivuori H, Loft A, Oldereid NB, Romundstad LB, Bergh C, Pinborg A. The health of children conceived by ART: ‘the chicken or the egg?’. Hum Reprod Update 2019;25:137–158. 30753453 10.1093/humupd/dmz001

[hoae041-B7] Bonduelle M , Wennerholm UB, Loft A, Tarlatzis BC, Peters C, Henriet S, Mau C, Victorin-Cederquist A, Van Steirteghem A, Balaska A et al A multi-centre cohort study of the physical health of 5-year-old children conceived after intracytoplasmic sperm injection, in vitro fertilization and natural conception. Hum Reprod 2005;20:413–419. 15576393 10.1093/humrep/deh592

[hoae041-B8] Carson C , Sacker A, Kelly Y, Redshaw M, Kurinczuk JJ, Quigley MA. Asthma in children born after infertility treatment: findings from the UK Millennium Cohort Study. Hum Reprod 2013;28:471–479. 23223378 10.1093/humrep/des398 PMC3545639

[hoae041-B9] Chambers GM , Dyer S, Zegers-Hochschild F, de Mouzon J, Ishihara O, Banker M, Mansour R, Kupka MS, Adamson GD. International Committee for Monitoring Assisted Reproductive Technologies world report: assisted reproductive technology, 2014^†^. Hum Reprod 2021;36:2921–2934. 34601605 10.1093/humrep/deab198

[hoae041-B10] Chen W , Peng Y, Ma X, Kong S, Tan S, Wei Y, Zhao Y, Zhang W, Wang Y, Yan L et al Integrated multi-omics reveal epigenomic disturbance of assisted reproductive technologies in human offspring. EBioMedicine 2020;61:103076. 33099088 10.1016/j.ebiom.2020.103076 PMC7585147

[hoae041-B11] China NHCotPsRo. Growth standard for children under 7 years of age. 2022. http://www.nhc.gov.cn/wjw/fyjk/202211/16d8b049fdf547978a910911c19bf389.shtml (26 June 2024, date last accessed).

[hoae041-B12] GBD Chronic Respiratory Disease Collaborators. Prevalence and attributable health burden of chronic respiratory diseases, 1990-2017: a systematic analysis for the Global Burden of Disease Study 2017. Lancet Respir Med 2020;8:585–596. 32526187 10.1016/S2213-2600(20)30105-3 PMC7284317

[hoae041-B13] Elhakeem A , Taylor AE, Inskip HM, Huang J, Tafflet M, Vinther JL, Asta F, Erkamp JS, Gagliardi L, Guerlich K et al; Assisted Reproductive Technology and Future Health (ART-Health) Cohort Collaboration. Association of Assisted Reproductive Technology With Offspring Growth and Adiposity From Infancy to Early Adulthood. JAMA Netw Open 2022;5:e2222106. 35881399 10.1001/jamanetworkopen.2022.22106 PMC9327583

[hoae041-B14] Ericson A , Nygren KG, Olausson PO, Kallen B. Hospital care utilization of infants born after IVF. Hum Reprod 2002;17:929–932. 11925384 10.1093/humrep/17.4.929

[hoae041-B15] Finnstrom O , Kallen B, Lindam A, Nilsson E, Nygren KG, Olausson PO. Maternal and child outcome after in vitro fertilization—a review of 25 years of population-based data from Sweden. Acta Obstet Gynecol Scand 2011;90:494–500. 21306346 10.1111/j.1600-0412.2011.01088.x

[hoae041-B16] Fruchter E , Beck-Fruchter R, Hourvitz A, Weiser M, Goldberg S, Fenchel D, Lerner-Geva L. Health and functioning of adolescents conceived by assisted reproductive technology. Fertil Steril 2017;107:774–780. 28093195 10.1016/j.fertnstert.2016.12.001

[hoae041-B17] Guibas GV , Moschonis G, Xepapadaki P, Roumpedaki E, Androutsos O, Manios Y, Papadopoulos NG. Conception via in vitro fertilization and delivery by Caesarean section are associated with paediatric asthma incidence. Clin Exp Allergy 2013;43:1058–1066. 23957341 10.1111/cea.12152

[hoae041-B18] Halliday J , Lewis S, Kennedy J, Burgner DP, Juonala M, Hammarberg K, Amor DJ, Doyle LW, Saffery R, Ranganathan S et al Health of adults aged 22 to 35 years conceived by assisted reproductive technology. Fertil Steril 2019;112:130–139. 31003618 10.1016/j.fertnstert.2019.03.001

[hoae041-B19] Hammad H , Lambrecht BN. The basic immunology of asthma. Cell 2021;184:1469–1485. 33711259 10.1016/j.cell.2021.02.016

[hoae041-B20] Hann M , Roberts SA, D’Souza SW, Clayton P, Macklon N, Brison DR. The growth of assisted reproductive treatment-conceived children from birth to 5 years: a national cohort study. BMC Med 2018;16:224. 30482203 10.1186/s12916-018-1203-7 PMC6260690

[hoae041-B21] Harju M , Keski-Nisula L, Raatikainen K, Pekkanen J, Heinonen S. Maternal fecundity and asthma among offspring-is the risk programmed preconceptionally? Retrospective observational study. Fertil Steril 2013;99:761–767.e1. 23148921 10.1016/j.fertnstert.2012.10.034

[hoae041-B22] Inhorn MC , Patrizio P. Infertility around the globe: new thinking on gender, reproductive technologies and global movements in the 21st century. Hum Reprod Update 2015;21:411–426. 25801630 10.1093/humupd/dmv016

[hoae041-B23] Kallen B , Finnstrom O, Nygren KG, Olausson PO. In vitro fertilization in Sweden: child morbidity including cancer risk. Fertil Steril 2005;84:605–610. 16169392 10.1016/j.fertnstert.2005.03.035

[hoae041-B24] Kallen B , Finnstrom O, Nygren KG, Olausson PO. Asthma in Swedish children conceived by in vitro fertilisation. Arch Dis Child 2013;98:92–96. 22875904 10.1136/archdischild-2012-301822

[hoae041-B25] Kuiper DB , Koppelman GH, la Bastide-van Gemert S, Seggers J, Haadsma ML, Roseboom TJ, Hoek A, Heineman MJ, Hadders-Algra M. Asthma in 9-year-old children of subfertile couples is not associated with in vitro fertilization procedures. Eur J Pediatr 2019;178:1493–1499. 31388755 10.1007/s00431-019-03436-2 PMC6733816

[hoae041-B26] Kuiper DB , Seggers J, Schendelaar P, Haadsma ML, Roseboom TJ, Heineman MJ, Hadders-Algra M. Asthma and asthma medication use among 4-year-old offspring of subfertile couples—association with IVF? Reprod Biomed Online 2015;31:711–714. 26380861 10.1016/j.rbmo.2015.08.002

[hoae041-B27] Leung TF , Ko FW, Sy HY, Tsui SK, Wong GW. Differences in asthma genetics between Chinese and other populations. J Allergy Clin Immunol 2014;133:42–48. 24188974 10.1016/j.jaci.2013.09.018

[hoae041-B28] Magnus MC , Karlstad Ø, Parr CL, Page CM, Nafstad P, Magnus P, London SJ, Wilcox AJ, Nystad W, Haberg SE. Maternal history of miscarriages and measures of fertility in relation to childhood asthma. Thorax 2019;74:106–113. 30514789 10.1136/thoraxjnl-2018-211886 PMC6467238

[hoae041-B29] Novakovic B , Lewis S, Halliday J, Kennedy J, Burgner DP, Czajko A, Kim B, Sexton-Oates A, Juonala M, Hammarberg K et al Assisted reproductive technologies are associated with limited epigenetic variation at birth that largely resolves by adulthood. Nat Commun 2019;10:3922. 31477727 10.1038/s41467-019-11929-9 PMC6718382

[hoae041-B30] Pandey S , Shetty A, Hamilton M, Bhattacharya S, Maheshwari A. Obstetric and perinatal outcomes in singleton pregnancies resulting from IVF/ICSI: a systematic review and meta-analysis. Hum Reprod Update 2012;18:485–503. 22611174 10.1093/humupd/dms018

[hoae041-B31] Polinski KJ , Stevens DR, Mendola P, Lin TC, Sundaram R, Bell E, Yeung EH. Infertility treatment associated with childhood asthma and atopy. Hum Reprod 2022;37:1609–1618. 35446387 10.1093/humrep/deac070 PMC9247411

[hoae041-B32] Qiao J , Chen Y, Yan LY, Yan J, Liu P, Sun QY. Changes in histone methylation during human oocyte maturation and IVF- or ICSI-derived embryo development. Fertil Steril 2010;93:1628–1636. 19394606 10.1016/j.fertnstert.2009.03.002

[hoae041-B33] Rosseel Y. lavaan: an R package for structural equation modeling. J Stat Soft 2012;48:1–36.

[hoae041-B34] Schaub AM , Gonzalez TL, Dorfman AE, Novoa AG, Hussaini RA, Harakuni PM, Khan MH, Shabani BJ, Swarna A, Wang ET et al A systematic review of genome wide analyses of methylation changes associated with assisted reproductive technologies in Various Tissues. Fertil Steril 2024;121:80–94. 37827482 10.1016/j.fertnstert.2023.10.007 PMC11262788

[hoae041-B35] Sicignano N , Beydoun HA, Russell H, Jones H, Oehninger S. A descriptive study of asthma in young adults conceived by IVF. Reprod Biomed Online 2010;21:812–818. 21050817 10.1016/j.rbmo.2010.07.009

[hoae041-B36] Song P , Adeloye D, Salim H, Dos Santos JP, Campbell H, Sheikh A, Rudan I. Global, regional, and national prevalence of asthma in 2019: a systematic analysis and modelling study. J Glob Health 2022;12:04052. 35765786 10.7189/jogh.12.04052 PMC9239324

[hoae041-B37] Tsabouri S , Lavasidis G, Efstathiadou A, Papasavva M, Bellou V, Bergantini H, Priftis K, Ntzani EE. Association between childhood asthma and history of assisted reproduction techniques: a systematic review and meta-analysis. Eur J Pediatr 2021;180:2007–2017. 33598756 10.1007/s00431-021-03975-7

[hoae041-B38] Tzeng CR , Huang Z, Asada Y, Zhang C, Ho MT, Li RHW, Kim JH, Govindarajan M, Vuyavanich T, Sini I et al Factors affecting the distribution of serum anti-müllerian hormone levels among infertile Asian women: a multi-nation, multi-centre, and multi-ethnicity prospective cohort study. Hum Reprod 2023;38:1368–1378. 37105234 10.1093/humrep/dead081

[hoae041-B39] Wijs LA , Doherty DA, Keelan JA, Penova-Veselinovic B, Burton P, Yovich JL, Hall GL, Sly PD, Holt PG, Hart RJ. Asthma and allergies in a cohort of adolescents conceived with ART. Reprod Biomed Online 2022;45:1255–1265. 36182641 10.1016/j.rbmo.2022.07.007

[hoae041-B40] Wijs LA , Fusco MR, Doherty DA, Keelan JA, Hart RJ. Asthma and allergies in offspring conceived by ART: a systematic review and meta-analysis. Hum Reprod Update 2021;28:132–148. 34642743 10.1093/humupd/dmab031

[hoae041-B41] Yang H , Ma Z, Peng L, Kuhn C, Rahmeh M, Mahner S, Jeschke U, von Schonfeldt V. Comparison of histone H3K4me3 between IVF and ICSI technologies and between boy and girl Offspring. Int J Mol Sci 2021;22:8574. 34445278 10.3390/ijms22168574 PMC8395251

[hoae041-B42] Zhang CX , Xue JL, Zhao W, Wu YQ, Liu XY, Wang SW, Li LH, Gu SM, Li JQ, Zhang YY et al Embryo morphologic quality in relation to the metabolic and cognitive development of singletons conceived by in vitro fertilization and intracytoplasmic sperm injection: a matched cohort study. Am J Obstet Gynecol 2022;227:479.e1–479.e23. 10.1016/j.ajog.2022.05.019 35568190

[hoae041-B43] Zhou Z , Zheng D, Wu H, Li R, Xu S, Kang Y, Cao Y, Chen X, Zhu Y, Xu S et al Epidemiology of infertility in China: a population-based study. BJOG 2018;125:432–441. 29030908 10.1111/1471-0528.14966

