# Supplementary material for: The risk of asthma in singletons conceived by ART: a retrospective cohort study
Source: Hum Reprod Open. 2024 Jun 19;2024(3):hoae041. doi: 10.1093/hropen/hoae041 (PMC11262460; doi:10.1093/hropen/hoae041)
Supplement: hoae041_Supplementary_Data [file hoae041_supplementary_data.zip › Supplementary-Tables final.docx]

Supplementary Table S1. Baseline characteristics of the unweighted sample, weighted sample and matched sample between ART-conceived offspring and NC offspring.

| Characteristics | Weighted ^a^ | | | Matched ^b^ | | |
| --- | --- | --- | --- | --- | --- | --- |
|  | NC | ART | SMD ^c^ | NC | ART | SMD ^c^ |
|  | (n=4619.6) | (n=4444.9) |  | (n=981) | (n=981) |  |
| Child Sex, No. (%) |  |  |  |  |  |  |
| Female | 2275.9(49.3) | 2103.9(47.3) | 0.039 | 481 (49.0) | 458 (46.7) | 0.047 |
| Male | 2343.7(50.7) | 2340.9(52.7) |  | 500 (51.0) | 523 (53.3) |  |
| Age at follow-up, median (IQR), years | 5(4-6) | 5(5-5) | 0.145^**^ | 5(4-5) | 5(4-5) | 0.046 |
| Maternal age, No. (%) |  |  |  |  |  |  |
| <35 | 3912.6 (84.7) | 3828.2 (86.1) | 0.041 | 860 (87.7) | 839 (85.5) | 0.063 |
| ≥35 | 707.0 (15.3) | 616.7 (13.9) |  | 121 (12.3) | 142 (14.5) |  |
| Paternal age, No. (%) |  |  |  |  |  |  |
| <35 | 3216.7 (69.6) | 3201.3 (72.0) | 0.053 | 757 (77.2) | 784 (79.9) | 0.067 |
| ≥35 | 1402.9 (30.4) | 1243.5 (28.0) |  | 224 (22.8) | 197 (20.1) |  |
| Maternal education level, No. (%) |  |  |  |  |  |  |
| <college | 3370.0 (72.9) | 3294.4 (74.1) | 0.026 | 717 (73.1) | 713 (72.7) | 0.009 |
| ≥college | 1249.6 (27.1) | 1150.5 (25.9) |  | 264 (26.9) | 268 (27.3) |  |
| Paternal education level, No. (%) |  |  |  |  |  |  |
| <college | 3377.5 (73.1) | 3215.8 (72.3) | 0.017 | 730 (74.4) | 713 (72.7) | 0.039 |
| ≥college | 1242.1 (26.9) | 1229.1 (27.7) |  | 251 (25.6) | 268 (27.3) |  |
| Maternal occupation type, No. (%) |  |  |  |  |  |  |
| Less advantage | 1604.3 (34.7) | 1539.2 (34.6) | 0.025 | 365 (37.2) | 376 (38.3) | 0.045 |
| Middle | 2294.9 (49.7) | 2249.4 (50.6) |  | 446 (45.5) | 451 (46.0) |  |
| Most advantaged | 720.3 (15.6) | 656.3 (14.8) |  | 170 (17.3) | 154 (15.7) |  |
| Paternal occupation type, No. (%) |  |  |  |  |  |  |
| Less advantage | 643.6 (13.9) | 656.6 (14.8) | 0.023 | 180 (18.3) | 198 (20.2) | 0.048 |
| Middle | 3201.5 (69.3) | 3063.0 (68.9) |  | 580 (59.1) | 572 (58.3) |  |
| Most advantaged | 774.5 (16.8) | 725.3 (16.3) |  | 221 (22.5) | 211 (21.5) |  |
| Maternal BMI, No. (%) |  |  |  |  |  |  |
| Normal | 3234.1 (70.0) | 3063.9 (68.9) | 0.064 | 673 (68.6) | 636 (64.8) | 0.095 |
| Underweight | 445.9 (9.7) | 445.7 (10.0) |  | 114 (11.6) | 124 (12.6) |  |
| Overweight | 793.3 (17.2) | 791.1 (17.8) |  | 169 (17.2) | 184 (18.8) |  |
| Obesity | 146.3 (3.2) | 144.2 (3.2) |  | 25 (2.5) | 37 (3.8) |  |
| Paternal BMI, No. (%) |  |  |  |  |  |  |
| Normal | 2529.0 (54.7) | 2351.5 (52.9) | 0.064 | 509 (51.9) | 492 (50.2) | 0.041 |
| Underweight | 115.1 (2.5) | 140.9 (3.2) |  | 31 (3.2) | 36 (3.7) |  |
| Overweight | 1613.5 (34.9) | 1548.5 (34.8) |  | 346 (35.3) | 357 (36.4) |  |
| Obese | 362.0 (7.8) | 404.0 (9.1) |  | 95 (9.7) | 96 (9.8) |  |
| Parental asthma, No. (%) | 186.3 (4.0) | 216.4 (4.9) | 0.040 | 34 (3.5) | 42 (4.3) | 0.042 |
| Smoking exposure, No. (%) | 1488.3 (32.2) | 1575.4 (35.4) | 0.068 | 414 (42.2) | 398 (40.6) | 0.033 |
| Residence, No. (%) |  |  |  |  |  |  |
| Rural | 2535.8 (54.9) | 2526.5 (56.8) | 0.039 | 695 (70.8) | 657 (67.0) | 0.084 |
| Urban | 2083.8 (45.1) | 1918.3 (43.2) |  | 286 (29.2) | 324 (33.0) |  |
| Duration of attempt to conceive, median (IQR), years | 0 (0-0) | 3(2-5) | 1.979^***^ | 0 (0-0) | 3(2-5) | 1.994^***^ |
| Previous live birth, No. (%) | 2079.9 (45.0) | 373.5 (8.4) | 0.909^***^ | 472 (48.1) | 78 (8.0) | 1.000^***^ |
| Fertilization methods, No. (%) |  |  |  |  |  |  |
| IVF | NA | 3318.9 (74.7) | NA | NA | 709 (72.3) | NA |
| ICSI | NA | 1125.9 (25.3) |  | NA | 272 (27.7) |  |
| Embryo transfer methods, No. (%) |  |  |  |  |  |  |
| Fresh | NA | 2685.8 (60.4) | NA | NA | 577 (58.8) | NA |
| Frozen | NA | 1759.0 (39.6) |  | NA | 404 (41.2) |  |
| Potential mediators |  |  |  |  |  |  |
| Obstetric complications, No. (%) | 1856.6 (40.2) | 2391.4 (53.8) | 0.275^***^ | 376 (38.3) | 518 (52.8) | 0.294^***^ |
| Gestational diabetes mellitus, No. (%) | 438.5 (9.5) | 443.2 (10.0) | 0.016 | 84 (8.6) | 104 (10.6) | 0.069 |
| Hypertensive disorder of pregnancy, No. (%) | 201.6 (4.4) | 236.0 (5.3) | 0.044 | 41 (4.2) | 53 (5.4) | 0.057 |
| Liver disorders in pregnancy, No. (%) | 100.8 (2.2) | 57.8 (1.3) | 0.068 | 19 (1.9) | 17 (1.7) | 0.015 |
| Premature rupture of the membranes, No. (%) | 58.5 (1.3) | 513.9 (11.6) | 0.43^***^ | 14 (1.4) | 114 (11.6) | 0.422^***^ |
| Respiratory distress syndrome, No. (%) | 54.0 (1.2) | 276.8 (6.2) | 0.271^***^ | 17 (1.7) | 65 (6.6) | 0.246^***^ |
| Cesarean section, No. (%) | 1914.9 (41.5) | 3521.2 (79.2) | 0.837^***^ | 393 (40.1) | 761 (77.6) | 0.824^***^ |
| Preterm birth, No. (%) | 326.9 (7.1) | 363.0 (8.2) | 0.041 | 67 (6.8) | 80 (8.2) | 0.05 |
| Macrosomia, No. (%) | 51.5 (1.1) | 258.6 (5.8) | 0.259^***^ | 15 (1.5) | 51 (5.2) | 0.205^***^ |
| Low birth weight, No. (%) | 270.4 (5.9) | 209.9 (4.7) | 0.051 | 64 (6.5) | 48 (4.9) | 0.07 |
| Neonatal intensive care unit admission, No. (%) | 640.5 (13.9) | 742.0 (16.7) | 0.079 | 124 (12.6) | 161 (16.4) | 0.107^*^ |
| Jaundice, No. (%) | 406.5 (8.8) | 707.5 (15.9) | 0.218^***^ | 76 (7.7) | 155 (15.8) | 0.252^***^ |
| Feeding patterns ^d^, No. (%) |  |  |  |  |  |  |
| Artificial only | 742.9 (16.1) | 809.8 (18.2) | 0.249^***^ | 168 (17.1) | 167 (17.0) | 0.285^***^ |
| Mixed | 1758.0 (38.1) | 1180.1 (26.6) |  | 383 (39.0) | 260 (26.5) |  |
| Breast only | 2118.7 (45.9) | 2454.9 (55.2) |  | 430 (43.8) | 554 (56.5) |  |
| BMI-for-age, No. (%) |  |  |  |  |  |  |
| Normal | 2858.2 (61.9) | 3585.1 (80.7) | 0.435^***^ | 585 (59.6) | 785 (80.0) | 0.484^***^ |
| Underweight | 507.3 (11.0) | 190.5 (4.3) |  | 95 (9.7) | 37 (3.8) |  |
| Overweight | 836.8 (18.1) | 489.8 (11.0) |  | 182 (18.6) | 121 (12.3) |  |
| Obese | 417.3 (9.0) | 179.5 (4.0) |  | 119 (12.1) | 38 (3.9) |  |

IPTW, inverse probability of treatment weighting; IQR, interquartile range; NA, not available; NC, naturally conceived; PSM, propensity score matching; SMD, standard mean difference.

^a^ IPTW analysis in which children were weighted by the inverse of the probability of being conceived by ART based on parental age(categorical), parental education level(categorical), parental BMI (categorical), parental occupation type (categorical), parental asthma (binary), smoking exposure (binary), residence type (binary), child sex (binary) and age at follow-up (continuous)

^b^ PSM analysis in which children were matched according to the same confounders as those included in IPTW, analysis at a 1:1 ratio, employing the nearest neighbor strategy with a caliper width of 0.2 and no replacement.

^c^ SMDs were shown to present the balance of the baseline characteristics between the two groups, before and after weighting and matching. *P* values were calculated using the χ2 test or Fisher exact test for categorical variables and the Mann-Whitney U tests for continuous variables. ^*^*P*<0.05, ^**^*P*<0.01, ^***^ *P*<0.001.

^d^ Feeding patterns in the first 6 months of life.

Supplementary Table S2. Missing characteristics before and after Imputation.

| Characteristics | Pre-imputation  (n=4433) | Post-imputation  (n=4433) | *P* value ^a^ |
| --- | --- | --- | --- |
| Paternal age, No. (%) |  |  |  |
| <35 | 3178 (71.9) | 3186 (71.9) | 1.000 |
| ≥ 35 | 1245 (28.1) | 1247 (28.1) |  |
| Maternal education, No. (%) |  |  |  |
| <college | 3299 (75.0) | 3329 (75.1) | 0.946 |
| ≥ college | 1099 (25.0) | 1104 (24.9) |  |
| Paternal education, No. (%) |  |  |  |
| <college | 3186 (72.5) | 3216 (72.5) | 0.959 |
| ≥college | 1210 (27.5) | 1217 (27.5) |  |
| Maternal occupation, No. (%) |  |  |  |
| Less advantage | 1516 (35.3) | 1564 (35.3) | 0.993 |
| Middle | 2204 (51.3) | 2271 (51.2) |  |
| Most advantaged | 576 (13.4) | 598 (13.5) |  |
| Paternal occupation, No. (%) |  |  |  |
| Less advantage | 645 (15.1) | 675 (15.2) | 0.946 |
| Middle | 3005 (70.1) | 3095 (69.8) |  |
| Most advantaged | 634 (14.8) | 663 (15.0) |  |
| Maternal BMI, No. (%) |  |  |  |
| Normal | 2924 (68.8) | 3059 (69.0) | 0.995 |
| Underweight | 419 (9.87) | 436 (9.84) |  |
| Overweight | 762 (17.9) | 794 (17.9) |  |
| Obese | 142 (3.34) | 144 (3.25) |  |
| Paternal BMI, No. (%) |  |  |  |
| Normal | 2124 (52.6) | 2336 (52.7) | 0.990 |
| Underweight | 126 (3.12) | 142 (3.20) |  |
| Overweight | 1421 (35.2) | 1548 (34.9) |  |
| Obese | 367 (9.09) | 407 (9.18) |  |
| Smoking exposure, No. (%) | 1507 (35.5) | 1577 (35.6) | 0.974 |
| Previous live birth, No. (%) | 821 (18.5) | 823 (18.6) | 0.994 |
| Obstetric complications, No. (%) | 2212 (50.0) | 2215 (50.0) | 1.000 |
| Gestational diabetes mellitus, No. (%) | 433 (9.90) | 437 (9.86) | 0.979 |
| Hypertensive disorders of pregnancy, No. (%) | 226 (5.18) | 228 (5.14) | 0.978 |
| Liver disorder in pregnancy, No. (%) | 66 (1.51) | 68 (1.53) | 1.000 |
| Premature rupture of the membranes, No. (%) | 381 (8.84) | 391 (8.82) | 0.999 |
| Respiratory distress syndrome, No. (%) | 220 (5.08) | 221 (4.99) | 0.874 |
| Cesarean section, No. (%) | 3022 (68.2) | 3027 (68.3) | 0.989 |
| Preterm, No. (%) | 352 (7.97) | 355 (8.01) | 0.982 |
| Macrosomia, No. (%) | 209 (4.83) | 210 (4.74) | 0.872 |
| Low birthweight, No. (%) | 233 (5.39) | 239 (5.39) | 1.000 |
| Neonatal intensive care unit admission, No. (%) | 696 (15.7) | 697 (15.7) | 1.000 |
| Feeding patterns ^b^, No. (%) |  |  |  |
| Artificial | 781 (17.8) | 791 (17.8) | 1.000 |
| Mixed | 1293 (29.5) | 1308 (29.5) |  |
| Breast | 2304 (52.6) | 2334 (52.7) |  |
| Body mass index-for age, No. (%) |  |  |  |
| Normal | 3285 (74.1) | 3308 (74.6) | 0.986 |
| Underweight | 247 (5.57) | 258 (5.82) |  |
| Overweight | 580 (13.1) | 591 (13.3) |  |
| Obese | 271 (6.11) | 276 (6.23) |  |
|  |  |  |  |

^a^ *P* values were calculated using the χ^2^ test or Fisher’s exact test for categorical variables and the Mann-Whitney U tests for continuous variables.

^b^ Feeding patterns in the first 6 months of life.

Supplementary Table S3. Baseline characteristics of ART-conceived offspring and NC offspring followed up during 2020-2023.

| Characteristic | NC  (n= 1206) | ART  (n= 680) | *P* value ^a^ |
| --- | --- | --- | --- |
| Child sex, No. (%) |  |  |  |
| Female | 580 (48.1) | 300 (44.1) | 0.107 |
| Male | 626 (51.9) | 380 (55.9) |  |
| Child age, years | 5 (4-5) | 5 (5-5) | 0.272 |
| Maternal age, | 27 (24-31) | 30 (28-33) | <0.001 |
| Paternal age | 29 (26-33) | 32 (29-35) | <0.001 |
| Maternal education, No. (%) |  |  |  |
| <college | 890 (73.8) | 449 (66.0) | <0.001 |
| ≥ college | 316 (26.2) | 231 (34.0) |  |
| Paternal education, No. (%) |  |  |  |
| <college | 898 (74.5) | 426 (62.6) | <0.001 |
| ≥ college | 308 (25.5) | 254 (37.4) |  |
| Maternal occupation, No. (%) |  |  |  |
| Less advantage | 471 (39.1) | 247 (36.3) | 0.059 |
| Middle | 532 (44.1) | 337 (49.6) |  |
| Most advantaged | 203 (16.8) | 96 (14.1) |  |
| Paternal occupation, No. (%) |  |  |  |
| Less advantage | 254 (21.1) | 62 (9.12) | <0.001 |
| Middle | 644 (53.4) | 525 (77.2) |  |
| Most advantaged | 308 (25.5) | 93 (13.7) |  |
| Maternal BMI, No. (%) |  |  |  |
| Normal | 782 (64.8) | 457 (67.2) | 0.032 |
| Underweight | 173 (14.3) | 66 (9.71) |  |
| Overweight | 221 (18.3) | 138 (20.3) |  |
| Obese | 30 (2.49) | 19 (2.79) |  |
| Paternal BMI, No. (%) |  |  |  |
| Normal | 615 (51.0) | 342 (50.3) | 0.735 |
| Underweight | 38 (3.15) | 18 (2.65) |  |
| Overweight | 442 (36.7) | 248 (36.5) |  |
| Obese | 111 (9.20) | 72 (10.6) |  |
| Residence, No. (%) |  |  |  |
| Rural | 901 (74.7) | 240 (35.3) | <0.001 |
| Urban | 305 (25.3) | 440 (64.7) |  |

| Characteristic | NC  (n= 1206) | ART  (n= 680) | *p* Value ^a^ |
| --- | --- | --- | --- |
| Parental asthma | 41 (3.40) | 55 (8.09) | <0.001 |
| Smoking exposure | 581 (48.2) | 209 (30.7) | <0.001 |
| Duration of attempt to conceive | 0.00 (0.;0) | 3 (2,5) | 0.000 |
| Previous live birth, No. (%) | 534 (44.3) | 75 (11.0) | <0.001 |
| Obstetric complications, No. (%) | 474 (39.3) | 309 (45.4) | 0.011 |
| Gestational diabetes mellitus, No. (%) | 94 (7.79) | 83 (12.2) | 0.002 |
| Hypertensive disorders of pregnancy, No. (%) | 47 (3.90) | 23 (3.38) | 0.659 |
| Liver disorder in pregnancy, No. (%) | 21 (1.74) | 8 (1.18) | 0.446 |
| Premature rupture of the membranes, No. (%) | 13 (1.08) | 74 (10.9) | <0.001 |
| Respiratory distress syndrome, No. (%) | 19 (1.58) | 28 (4.12) | 0.001 |
| Cesarean section, No. (%) | 463 (38.4) | 443 (65.1) | <0.001 |
| Preterm, No. (%) | 77 (6.38) | 56 (8.24) | 0.157 |
| Macrosomia, No. (%) | 18 (1.49) | 39 (5.74) | <0.001 |
| Low birthweight, No. (%) | 78 (6.47) | 32 (4.71) | 0.143 |
| Neonatal intensive care unit admission, No. (%) | 149 (12.4) | 165 (24.3) | <0.001 |
| Jaundice, No. (%) | 92 (7.63) | 130 (19.1) | <0.001 |
| Feeding patterns ^b^, No. (%) |  |  |  |
| Artificial | 201 (16.7) | 97 (14.3) | <0.001 |
| Mixed | 463 (38.4) | 136 (20.0) |  |
| Breast | 542 (44.9) | 447 (65.7) |  |
| BMI-for-age, No. (%) |  |  |  |
| Normal | 712 (59.0) | 567 (83.4) | <0.001 |
| Underweight | 110 (9.12) | 16 (2.35) |  |
| Overweight | 234 (19.4) | 81 (11.9) |  |
| Obese | 150 (12.4) | 16 (2.35) |  |

NC, naturally conceived

^a^ *P* values were calculated using the χ2 test, Fisher’s exact test or Mann-Whitney U tests.

^b^ Feeding patterns in the first 6 months of life.

Supplementary Table S4. Asthma in ART-conceived offspring and NC offspring followed up during 2020-2023.

| Analysis | NC  (n= 1206) | ART  (n= 680) |
| --- | --- | --- |
| Asthma, No. (%) | 51 (4.23) | 27 (397) |
| Crude analysis, OR (95%CI) | Ref. | 0.94 (0.57-1.49) |
| Multivariable analysis ^a^, aOR (95%CI) | Ref. | 0.98 (0.30-2.95) |

aOR, adjusted odds ratio; NC, naturally conceived; OR, odds ratio; Ref., Reference.

^a^ Adjusted for parental age (continous), parental education level(categorical), parental BMI (categorical), parental occupation type (categorical), parental asthma (binary), smoking exposure (binary), residence type (binary), child sex (binary) and age at follow-up (continuous) with multivariable analysis using logistics regression model.

Supplementary Table S5. Stratified and interaction analyses in associations between asthma in children and ART.

| Subgroups | aOR (95% CI) ^a^ | *P* value for interaction ^a^ |
| --- | --- | --- |
| Duration of attempt to conceive |  |  |
| <1 | 1.01(0.20-4.07) | 0.567 |
| ≥ 1 | 1.31(0.40-8.08) |  |
| Child Sex |  |  |
| Female | 0.87(0.5-1.53) | 0.350 |
| Male | 1.26(0.8-2.02) |  |
| Maternal age |  |  |
| <35 | 0.94(0.65-1.36) | 0.061 |
| ≥ 35 | 4.44(1.19-29.09) |  |
| Paternal age |  |  |
| <35 | 0.61(0.33-1.11) | 0.980 |
| ≥ 35 | 1.12(0.57-2.35) |  |
| Maternal education |  |  |
| <college | 1.50(0.97-2.40) | 0.087 |
| ≥ college | 0.59(0.32-1.07) |  |
| Paternal education |  |  |
| <college | 1.28(0.83-2.01) | 0.289 |
| ≥ college | 0.76(0.41-1.44) |  |
| Maternal occupation |  |  |
| Less advantage | 1.25(0.70-2.32) | 0.649 |
| Middle | 1.10(0.66-1.88) |  |
| Most advantaged | 0.67(0.28-1.68) |  |
| Paternal occupation |  |  |
| Less advantage | 0.78(0.29-2.1) | 0.380 |
| Middle | 1.30(0.81-2.18) |  |
| Most advantaged | 0.86(0.44-1.68) |  |
| Maternal BMI |  |  |
| Normal | 1.07(0.70-1.66) | 0.368 |
| Underweight | 0.85(0.15-4.65) |  |
| Overweight | 1.00(0.30-3.28) |  |
| Obese | -^b^ |  |
| Paternal BMI |  |  |
| Normal | 1.08(0.67-1.8) | 0.651 |
| Underweight | 0.27(0.01-5.63) |  |
| Overweight | 1.41(0.75-2.8) |  |
| Obese | 0.59(0.21-1.74) |  |
| Parental asthma |  |  |
| No | 1.04(0.73-1.51) | 0.614 |
| Yes | 2.58(0.52-18.67) |  |

aOR, adjusted odds ratio

^a^ Stratified and interaction analyses were conducted with multivariable logistic regression, and models were adjusted for the following covariates except for stratified variables: parental age, parental education level, parental body mass index, parental occupation type, parental asthma, smoking exposure, residence type, child sex and age at follow-up.

^b^ The sample size was too small to compute.

Supplementary Table S6. Mediating effect and proportions of obstetrical and neonatal outcome between ART and childhood asthma.

| Pathways ^a^ | Indirect  effect (%) | 95% CI | | *P* Value | Direct  Effect(%) | 95% CI | | *P* Value | Mediation  Proportion(%) |
| --- | --- | --- | --- | --- | --- | --- | --- | --- | --- |
| **ART→ Obstetric complications→ Asthma** | **0.29** | **0.04** | **0.48** | **0.012** | 0.02 | 0.04 | 1.51 | 0.996 | 64.49 |
| ART→ Gestational diabetes mellitus→ Asthma | 0.00 | -0.03 | 0.08 | 0.668 | 0.24 | -0.03 | 1.72 | 0.78 | -1.83 |
| ART→ Hypertensive disorders of pregnancy→ Asthma | 0.01 | -0.03 | 0.09 | 0.648 | 0.24 | -0.03 | 1.71 | 0.792 | 3.03 |
| ART→ Liver disorder in pregnancy→ Asthma | 0.00 | -0.04 | 0.06 | 0.76 | 0.25 | -0.04 | 1.70 | 0.784 | 1.92 |
| ART→ Premature rupture of the membranes→Asthma | 0.06 | -0.18 | 0.33 | 0.692 | 0.19 | -0.18 | 1.58 | 0.816 | 22.29 |
| ART→ Respiratory distress syndrome→ Asthma | 0.09 | -0.06 | 0.26 | 0.236 | 0.16 | -0.06 | 1.63 | 0.876 | 35.87 |
| ART→ Cesarean section→ Asthma | 0.31 | -0.22 | 0.81 | 0.24 | -0.03 | -0.22 | 1.53 | 0.984 | 72.62 |
| ART→ Preterm birth→ Asthma | 0.03 | -0.06 | 0.21 | 0.288 | 0.20 | -0.06 | 1.65 | 0.808 | 11.63 |
| ART→ Macrosomia→ Asthma | 0.05 | -0.09 | 0.23 | 0.496 | 0.20 | -0.09 | 1.66 | 0.856 | 20.32 |
| ART→ Low birth weight→ Asthma | -0.08 | -0.17 | 0.01 | 0.136 | 0.31 | -0.17 | 1.74 | 0.696 | -35.30 |
| ART→ Jaundice→ Asthma | 0.10 | -0.05 | 0.31 | 0.204 | 0.16 | -0.05 | 1.64 | 0.868 | 38.50 |
| **ART→ Neonatal intensive care unit admission→ Asthma** | **0.08** | **0.01** | **0.26** | **0.024** | 0.17 | 0.01 | 1.65 | 0.856 | 32.11 |
| **ART→ Feeding pattern→ Asthma** ^b^ | **-0.19** | **-0.30** | **-0.03** | **0.004** | 0.36 | -0.30 | 1.80 | 0.684 | -32.64 |
| ART→ BMI-for-age→ Asthma | -0.04 | -0.18 | 0.08 | 0.5 | 0.30 | -0.18 | 1.78 | 0.728 | -16.16 |

^a^ R package Mediation was used to calculate the indirect and direct effect of pathways and analyses were adjusted for parental age (continuous), parental education level(categorical), parental BMI (categorical), parental occupation type (categorical), parental asthma (binary), smoking exposure (binary) residence type (binary), child sex (binary), age at follow-up (continuous) and year at follow-up (categorical). Statistical significance was set at *P*<0.05.

^b^ Feeding pattern was included as an ordered factor and we assigned 1 for artificial feeding, 2 for mixed feeding, and 3 for breastfeeding.
