# Supplementary figures and images for: The risk of asthma in singletons conceived by ART: a retrospective cohort study
Source: Hum Reprod Open. 2024 Jun 19;2024(3):hoae041. doi: 10.1093/hropen/hoae041 (PMC11262460; doi:10.1093/hropen/hoae041)

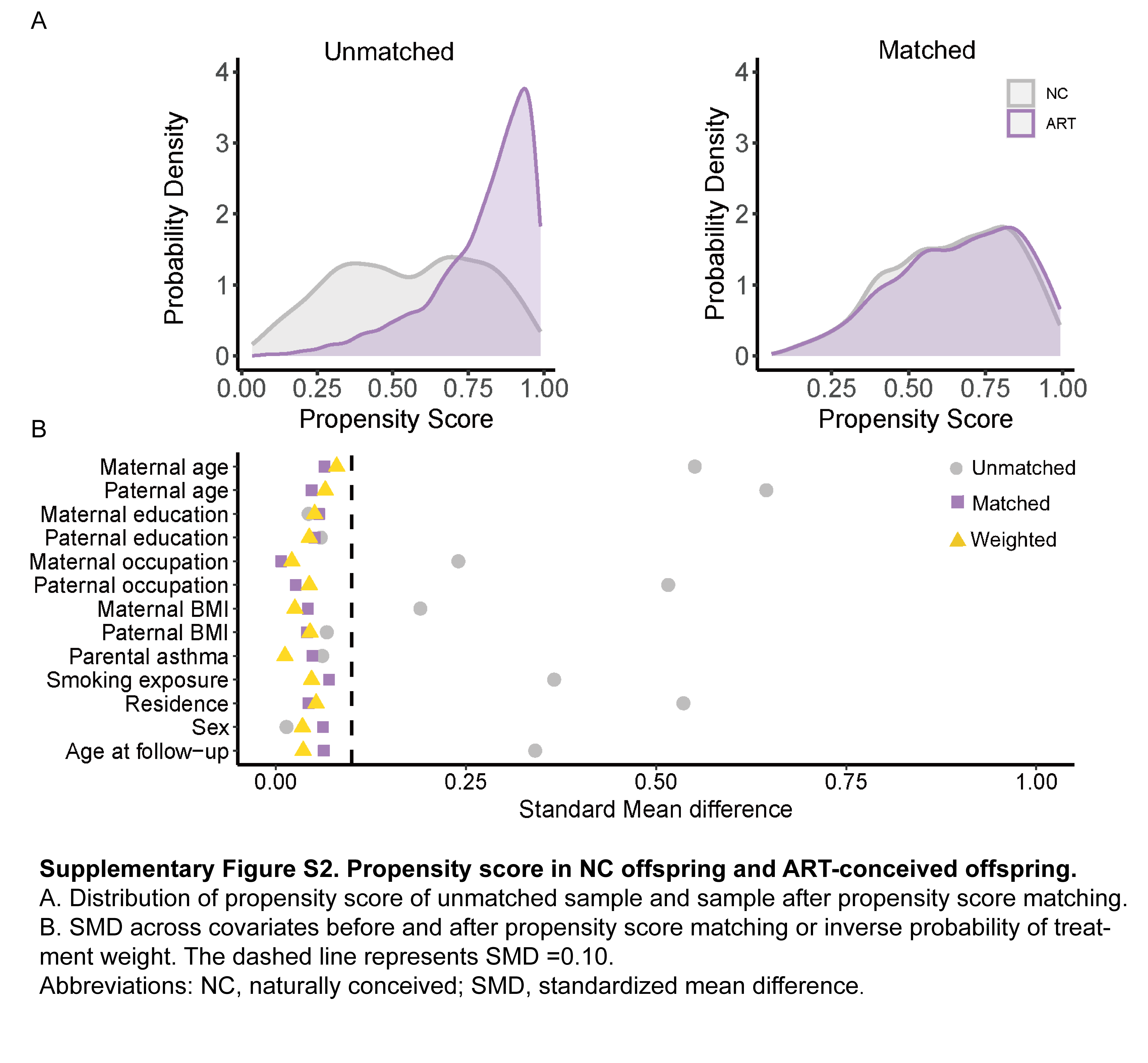

Supplement: hoae041_Supplementary_Data [file hoae041_supplementary_data.zip › Supplementary Figure S2 final.tif]
